# Supplementary material for: Dimensions of music use motivations: Genetic and environmental underpinnings, and associations with Big Five and Empathy traits
Source: PLoS One. 2025 Aug 8;20(8):e0329808. doi: 10.1371/journal.pone.0329808 (PMC12334019; doi:10.1371/journal.pone.0329808)
Supplement: S1 File — S1 Table. Music use motivation questionnaire. Item and subscale descriptive statistics of the music use motivation scale. S2 Table. Tests of acquiescence effects in the music use motivations variables. S3 Table. Tests of the assumptions of equal means and variances across twin order and zygosity in a multivariate model of the four music use motivations variables. Models 2–4 are compared with the fully saturated model 1, showing no significant effects of constraining means and variances (all p’s > .130). S4 Table. Tests of the assumptions of equal means and variances across twin order and zygosity in a multivariate model of the four empathy facets. Models 2–4 are compared with the fully saturated model 1, showing no significant effects of constraining means and variances (all p’s > .348). S5 Table. Tests of the assumptions of equal means and variances across twin order and zygosity in univariate models of the Big Five facets that were included in the final twin models. Models 2–4 are compared with the fully saturated model 1, showing no significant effects of constraining means and variances (all p’s > .061). S6 Table. Model fitting results for the multivariate biometric models of the four music use motivation dimensions. S7 Table. Pairwise phenotypic correlations among music use motivation subscales and the broad personality domains. S8 Table. Pairwise phenotypic correlations among all study variables. S9 Table. Model fitting results from the multivariate biometric models of the covariance between the music use motivation subscales and personality facets. S10 Table. Model estimates derived from the best-fitting AE model of the MM-transcendence – personality associations. Standardized genetic (A) and unique environmental (E) path estimates, as well as genetic (rA) and unique environmental (rE) correlations. S11 Table. Model estimates derived from the best-fitting AE model of the MM-emotion regulation – personality associations. Standardized genetic (A) and unique env [file pone.0329808.s001.zip › supplementary materials/Table_S1.docx]

**Table S1: Music use motivation questionnaire.**

Item and subscale descriptive statistics of the music use motivation scale.

| Subscales and items | Item# | Mean | SD | Skew | Kurtosis |
| --- | --- | --- | --- | --- | --- |
| *Musical Transcendence* |  | 2.83 | 0.85 | -0.11 | -0.37 |
| I seek deep experiences through music | 1 | 2.65 | 1.11 | 0.17 | -0.75 |
| Music raises me to another state of mind | 7 | 3.35 | 1.1 | -0.67 | -0.27 |
| I feel music communicates what language can’t | 9 | 3.17 | 1.12 | -0.36 | -0.55 |
| I like to use music for the very intense experience it gives me | 12 | 2.72 | 1.11 | 0.06 | -0.76 |
| Music exposes me to emotions I don’t often feel | 16 | 2.73 | 1.05 | 0.02 | -0.70 |
| Music inspires new ideas and thoughts in me | 18 | 2.97 | 1.05 | -0.14 | -0.66 |
| Music helps me understand who I am | 19 | 2.65 | 1.07 | 0.1 | -0.73 |
| Music listening sparks my creativity | 23 | 2.98 | 1.14 | -0.24 | -0.81 |
| Music helps me discover who I want to be | 25 | 2.28 | 1.03 | 0.42 | -0.55 |
| Music is like a comforting friend to me | 28 | 2.79 | 1.21 | -0.01 | -1.03 |
|  |  |  |  |  |  |
| *Emotion Regulation* |  | 2.62 | 0.91 | 0.12 | -0.59 |
| I like to use music to distract me from my worries | 4 | 2.84 | 1.19 | -0.04 | -1.04 |
| I use music to distract me from physical aches | 6 | 2.07 | 1.07 | 0.81 | -0.14 |
| I listen or play music when I’m upset or feeling down | 13 | 3.15 | 1.16 | -0.36 | -0.75 |
| I use music to calm myself when I’m stressed or feeling anxious | 15 | 2.94 | 1.18 | -0.14 | -0.98 |
| I use music to help me work through my emotional problems | 20 | 2.73 | 1.18 | 0.08 | -1.03 |
| I use music to explore and understand my own feelings | 22 | 2.38 | 1.07 | 0.41 | -0.54 |
| I use music to distract me from emotional pain | 24 | 2.34 | 1.15 | 0.5 | -0.71 |
| I use music to get through difficult times | 27 | 2.71 | 1.22 | 0.08 | -1.09 |
| Playing music is an outlet for my anger or frustrations | 30 | 2.43 | 1.16 | 0.36 | -0.90 |
|  |  |  |  |  |  |
| *Social* |  | 2.80 | 0.70 | -0.27 | -0.09 |
| Concerts often make me feel part of a community | 2 | 3.28 | 1.07 | -0.56 | -0.32 |
| Having similar taste in music often helps me relate better to my peers | 3 | 2.7 | 1.04 | 0 | -0.75 |
| I like to listen to music that my friends like | 10 | 3 | 0.87 | -0.45 | 0.01 |
| Music is important for informing and maintaining relationships | 11 | 2.66 | 0.99 | -0.1 | -0.64 |
| I often use music to feel a closer bond with other people | 14 | 2.42 | 0.97 | 0.22 | -0.53 |
| Music helps me feel comfortable around other people | 26 | 2.54 | 1.09 | 0.1 | -0.94 |
| Music is more powerful when I experience it with others | 29 | 2.98 | 1.07 | -0.3 | -0.64 |
|  |  |  |  |  |  |
| *Music identity and expression* |  | 2.93 | 0.88 | -0.07 | -0.46 |
| I consider myself a music 'fan' or music buff of certain types of music | 5 | 2.88 | 1.23 | 0.04 | -0.99 |
| I feel safe expressing my feelings through music | 8 | 2.87 | 1.11 | -0.08 | -0.70 |
| My music collection/playlist says a lot about me | 17 | 3.34 | 1 | -0.5 | -0.19 |
| I dance, sing or play music to express my feelings | 21 | 2.65 | 1.18 | 0.15 | -1.01 |

*Abbreviations. SD* Standard Deviation.
