# Supplementary material for: Dimensions of music use motivations: Genetic and environmental underpinnings, and associations with Big Five and Empathy traits
Source: PLoS One. 2025 Aug 8;20(8):e0329808. doi: 10.1371/journal.pone.0329808 (PMC12334019; doi:10.1371/journal.pone.0329808)
Supplement: S1 File — S1 Table. Music use motivation questionnaire. Item and subscale descriptive statistics of the music use motivation scale. S2 Table. Tests of acquiescence effects in the music use motivations variables. S3 Table. Tests of the assumptions of equal means and variances across twin order and zygosity in a multivariate model of the four music use motivations variables. Models 2–4 are compared with the fully saturated model 1, showing no significant effects of constraining means and variances (all p’s > .130). S4 Table. Tests of the assumptions of equal means and variances across twin order and zygosity in a multivariate model of the four empathy facets. Models 2–4 are compared with the fully saturated model 1, showing no significant effects of constraining means and variances (all p’s > .348). S5 Table. Tests of the assumptions of equal means and variances across twin order and zygosity in univariate models of the Big Five facets that were included in the final twin models. Models 2–4 are compared with the fully saturated model 1, showing no significant effects of constraining means and variances (all p’s > .061). S6 Table. Model fitting results for the multivariate biometric models of the four music use motivation dimensions. S7 Table. Pairwise phenotypic correlations among music use motivation subscales and the broad personality domains. S8 Table. Pairwise phenotypic correlations among all study variables. S9 Table. Model fitting results from the multivariate biometric models of the covariance between the music use motivation subscales and personality facets. S10 Table. Model estimates derived from the best-fitting AE model of the MM-transcendence – personality associations. Standardized genetic (A) and unique environmental (E) path estimates, as well as genetic (rA) and unique environmental (rE) correlations. S11 Table. Model estimates derived from the best-fitting AE model of the MM-emotion regulation – personality associations. Standardized genetic (A) and unique env [file pone.0329808.s001.zip › supplementary materials/Table_S4.docx]

**Table S4:** **Tests of the assumptions of equal means and variances across twin order and zygosity in a multivariate model of the four empathy facets.**

Models 2-4 are compared with the fully saturated model 1, showing no significant effects of constraining means and variances (all p’s > .348).

**
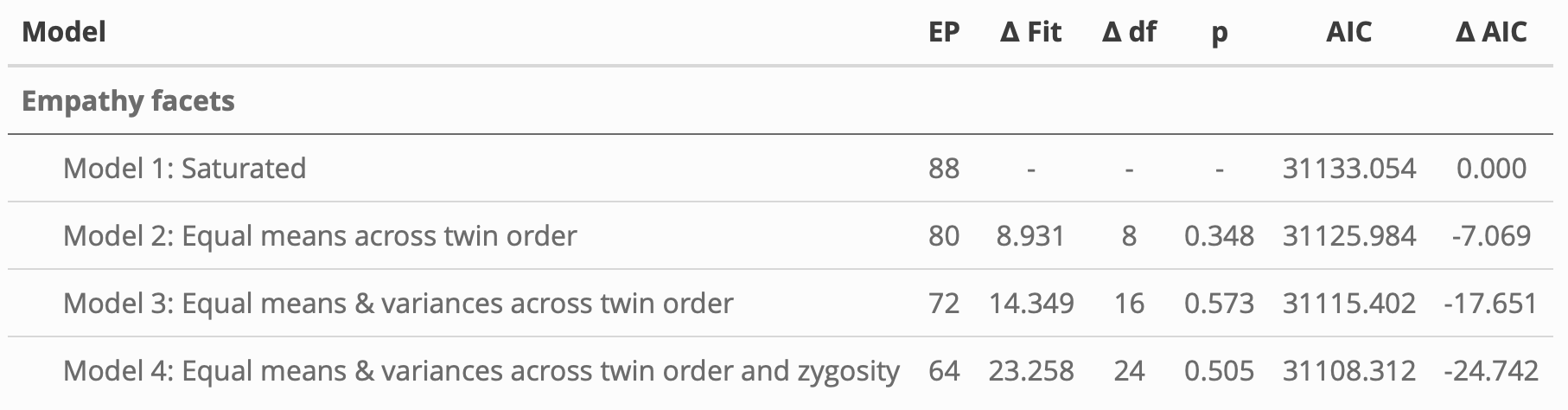
**
